# Perceived barriers to facemask adherence in the covid-19 pandemic in Pakistan-A cross-sectional survey

**DOI:** 10.1371/journal.pone.0267376

**Published:** 2022-05-19

**Authors:** Khadijah Abid, Hassan Ahmed, Yashfika Abdul Bari, Maryam Younus, Zainab Pervez Khambati, Abira Imran, Abdul Jabbar

**Affiliations:** 1 Department of Public Health, Shaheed Zulfikar Ali Bhutto Institute of Science and Technology, Karachi, Sindh, Pakistan; 2 Oral Sciences, University of Glasgow, Glasgow, United Kingdom; 3 Ministry of Finance, Trade and Development Authority of Pakistan, Karachi, Sindh, Pakistan; 4 Medicine Department, Jinnah Medical and Dental College, Karachi, Sindh, Pakistan; 5 Biostatistics Department, Liaquat National Hospital, Karachi, Sindh, Pakistan; 6 Clinical Medicine, University of Veterinary and Animal Sciences, Lahore, Punjab; Jouf University, Kingdom of Saudi Arabia, SAUDI ARABIA

## Abstract

**Objective:**

To explore perceived barriers associated with facemask adherence to prevent spread of COVID-19 spread in Pakistani population.

**Methodology:**

A cross sectional study was conducted from 25-July 2020 to 5-August 2020. Participants of both genders of age >17 years, currently residing in Pakistan, who had access to internet and understood English were included in the survey. The survey was designed on Google form and was distributed digitally across different areas of Pakistan via social media. Survey included questions regarding socio-demographics, facemask adherence and perceived barriers related to facemask adherence such as perceived risks, health concerns, comfort, social influences, religious/cultural norms and social protocols and health recommendations. SPSS version 23 was used to analyze data. Independent t-test/One-way ANOVA was applied to assess significant difference between perceived barriers to wear face mask and socio-demographic factors, p-value ≤0.05 was taken as statistically significant. Post-hoc LSD test was also applied where applicable.

**Results:**

Only 20% of the participants reported non-adherence to facemask. Amongst these participants, majority agreed that comfort was the main barrier precluding them from wearing a mask, 89.4% subjects saying that it was too hot to wear it and 84.1% saying that a mask was too uncomfortable to wear. Whereas, 82.1% highly agreed that difficulty in breathing is perceived barrier related to facemask usage. Statistically significant difference was found between health concerns with gender (p = 0.031), locality (p = 0.001) and religion (p = 0.03); comfort with locality (p = 0.007); social influences with gender (p = 0.001), ethnicity (p = 0.001) and locality (p = 0.017); cultural/religious norms with religion (p = 0.001) and social protocols and health recommendations with age (p = 0.015).

**Conclusion:**

Despite of satisfactory facemask adherence, still there are perceived barriers to it. In order to increase utilization of face masks among the general population, strict health policies should be implemented and awareness regarding the importance of face masks should be enhanced by educational interventions.

## Introduction

COVID-19 is a respiratory illness that emerged in China in December 2019 and spread widely around the world [1]. As of 26 July 2020, 15,785,641 confirmed cases of COVID-19, including 640,016 fatalities, have been reported to the WHO worldwide [2]. Evidence suggests that the coronavirus is predominantly spread from person to person through respiratory droplets and contact [3]. Globally, one in five individuals might have higher odds of developing severe COVID-19 due to underlying diseases. However, this probability differs considerably by age, gender, ethnicity, socio-economic status, occupation and education level [4].

The public health initiatives including closure of border, cancellation of flights, quarantine, massive testing for case detection, rapid contact tracing, regular hand washing, sanitization of materials and subsequent social distancing measures comprising of closure of school, work from home, cancelation of all large gatherings and social activities excluding essential services were also adopted to various degrees and for specific periods in different geographical areas to minimize the possibility of community transmission [5]. In past many of these initiatives were adopted to stop the spread of severe respiratory syndromes (SARS) and pandemic influenza A H1N1 in China and other countries [6, 7].

Previous studies showed that community-wide use of facemask during the past epidemics or current pandemic of COVID-19 can be effective in breaking the chain of transmission of the virus. In Beijing a case-control study was carried out during SARS 2003, which showed 70% reduction in risk of SARS 2003 transmission by wearing face mask outdoors [8]. WHO and other health organizations also agreed to the fact that facemask can limit COVID-19 spread in communities [9–11]. However, use of facemask remains controversial among general population, due to different guidelines by governments and public health organizations initially disagreeing on the guidelines for wearing facemask [5]. According to recent research, the most common reported hurdles to facemask adherence were discomfort, financial reasons, and forgetfulness [12]. Additionally, other studies reported health literacy, a lack of resources, and poverty were the most significant hurdles to COVID-19 prevention [13–16].

In Pakistan, outbreak of COVID-19 poses a significant burden to healthcare facilities. Government of Pakistan has made the use of face masks compulsory in public and crowded places on 30 May 2020 [17]. Although the Pakistani government is actively promoting face masks, hand washing and sanitization, and social distance, many individuals remain hesitant to take these measures. There are segments of the people in Pakistan who do not understand how and why facemasks can protect them from COVID-19. Hence, the current study was conducted to explore perceived barriers associated with facemask usage among the general population to prevent COVID-19 spread in Pakistan. Addressing these barriers and their associated factors paves the way for strategies that support widespread use of mask, and enable policy makers to make decisions with a focus on risk perceptions, thus aiding in Covid-19 prevention.

## Methodology

It was a cross-sectional survey conducted from 25-July 2020 to 5-August 2020. Sample size of 596≈600 was estimated using PASS version 11 sample size calculator, by taking statistics as 79.8% for usage of facemask among general public in Pakistan [18], margin of error as 3.3% and 95% confidence level. Subjects of age >17 years, of either gender, currently residing in Pakistan, who had access to internet, and understood English were included in the survey. Survey was distributed digitally across different areas of Pakistan via social media i.e. Facebook, Gmail, LinkedIn, Instagram, Snapchat, Twitter and WhatsApp. Non-random convenience sampling technique was employed for selecting the subjects. Participants with known and diagnosed respiratory disorders and who did not give consent were excluded from the study.

Survey was designed on google forms in English language. Survey included an informed consent form in which participants were informed of the objectives, risks, and benefits of the study. Participation in the survey was totally voluntary and all information was kept secure and confidential. Participants were free to withdraw from the study at any point.

**Fig 1:**
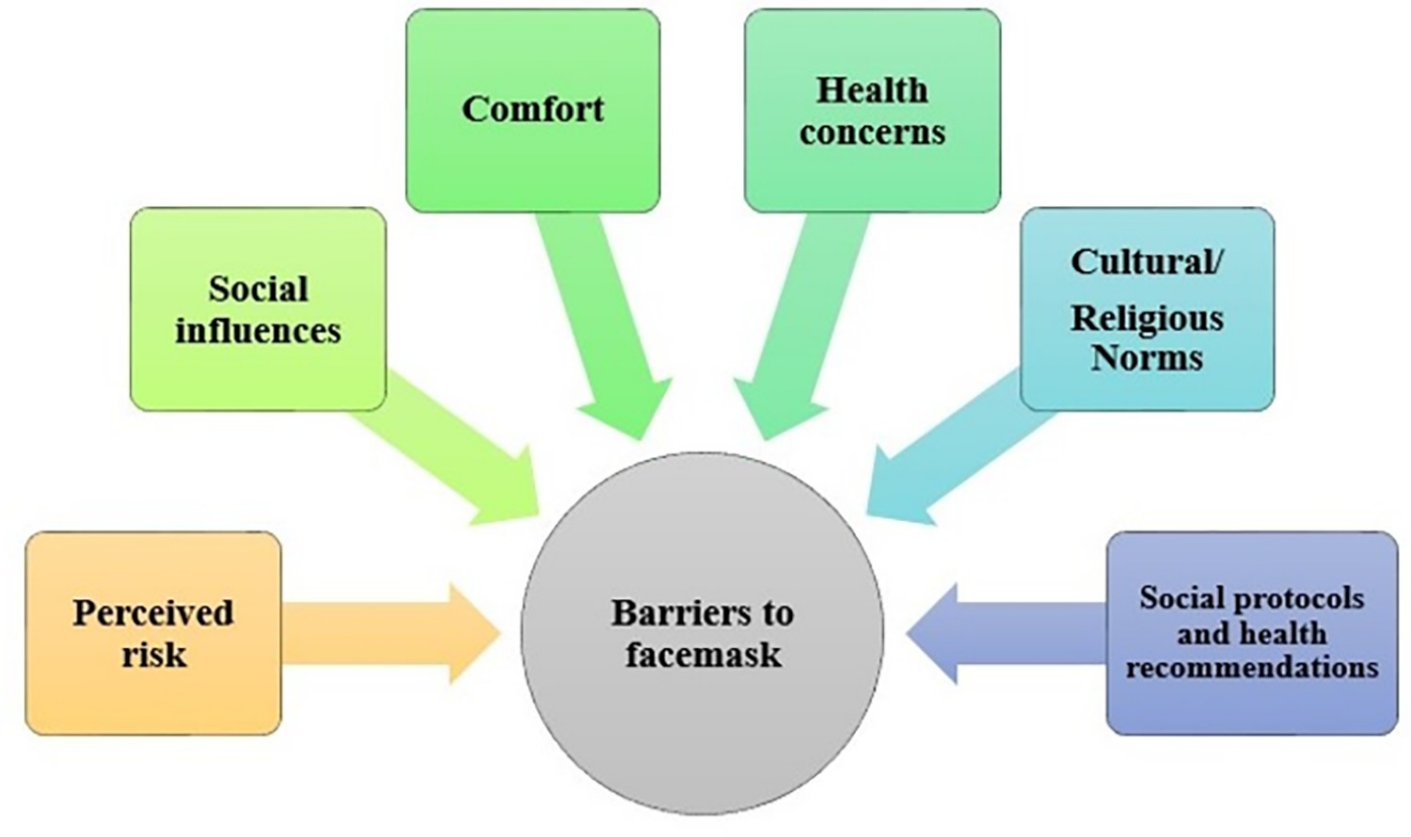
Conceptual framework for perceived barriers for facemask adherence.

The survey included 31 questions. There were questions regarding socio-demographics such as age, gender, locality, ethnicity, education level, employment status and marital status. There was a question regarding adherence to facemask i.e. “Do you wear a facemask when you go outside or in public/crowd to prevent/limit COVID-19 spread?” with responses as ‘yes’ or ‘no’. The participants who answered “no” were then asked for perceived barriers related to wearing a facemask i.e. (1) perceived risk, (2) health concerns, (3) comfort, (4) social influences, (5) cultural/religious norms and (6) social protocols and health recommendations [19]. (Fig 1) Three items were designed for perceived risks to assess the extent to which responders perceive they are at risk of getting the COVID-19. Three items were designed for health concerns to assess the extent to which respondents perceive they face health issues when they wear a facemask. Four items were used to assess opinions of responders regarding comfort of facemask. Seven items were used to see if social influences were perceived barriers for wearing a face mask for COVID-19 prevention. One item was to assess the religious or cultural barriers to wearing face masks to prevent the spread of COVID-19. Two items were designed to assess if health policy or recommendations from health consultants, were one of the barriers influencing the respondents. Participants were asked to rate how much they perceived each barrier on a 5-point Likert scale [1 = Strongly disagree (SD), 2 = Disagree (D), 3 = Neutral (N), 4 = Agree (A) and 5 = Strongly agree (SA)]. We calculated mean composite score of each perceived barrier by adding individual’s score and dividing it by the number of items constituting the sub-scale. Following this, participants were asked the question “In their opinion what is the best alternative for a facemask”. Estimated time to complete the whole survey was 10–15 minutes per person.

Pilot testing of questionnaire was done using reliability analysis. For pilot testing 30 samples were included in the analysis according to a flat rule of thumb [20] before administration and Cronbach’s alpha value was estimated as 71%.

This research followed ethical guidelines as specified by Declaration of Helsinki. Ethical approval was sought by ethical review committee of Ameen Medical and Dental Center, Karachi Pakistan (Ref# ERC-AMDC/016/2020).

SPSS version 23 was used to analyze data. Mean and SD were computed for numeric variable like age and score of perceived barriers (sub-scales). Frequency and percentages were estimated for categorical variables like age groups, gender, ethnicity, locality, religion, education level, employment status and marital status. Independent t-test/One-way ANOVA was applied to assess significant difference between perceived barriers to wear face mask and potential factors i.e. age, gender, ethnicity, locality, religion, education level, employment status and marital status, p-value ≤0.05 was taken as statistically significant. Post-hoc LSD test was also applied where applicable.

## Results

**Fig 2:**
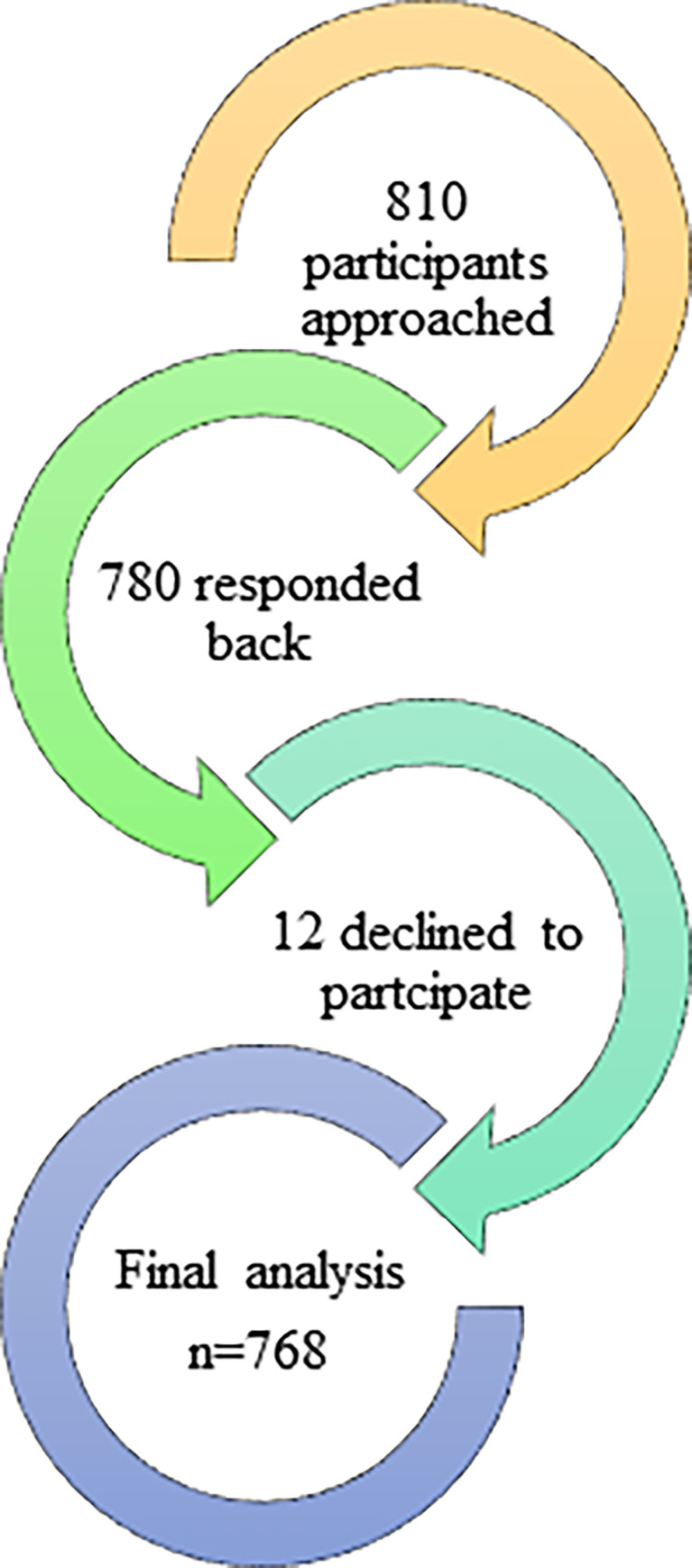
Flowchart of study participants.

After inflating the sample size by 30% for non-respondents, a total of 810 participants were approached. The survey required participants to fill out all of the questions with no omissions allowed, so they were no missing values in the data. Out of 810 participants, 780 participants responded back (response rate = 96.2%). However, 12 participants declined to participate in the survey and thus in the final analysis 768 participants were included (Fig 2).

**Fig 3:**
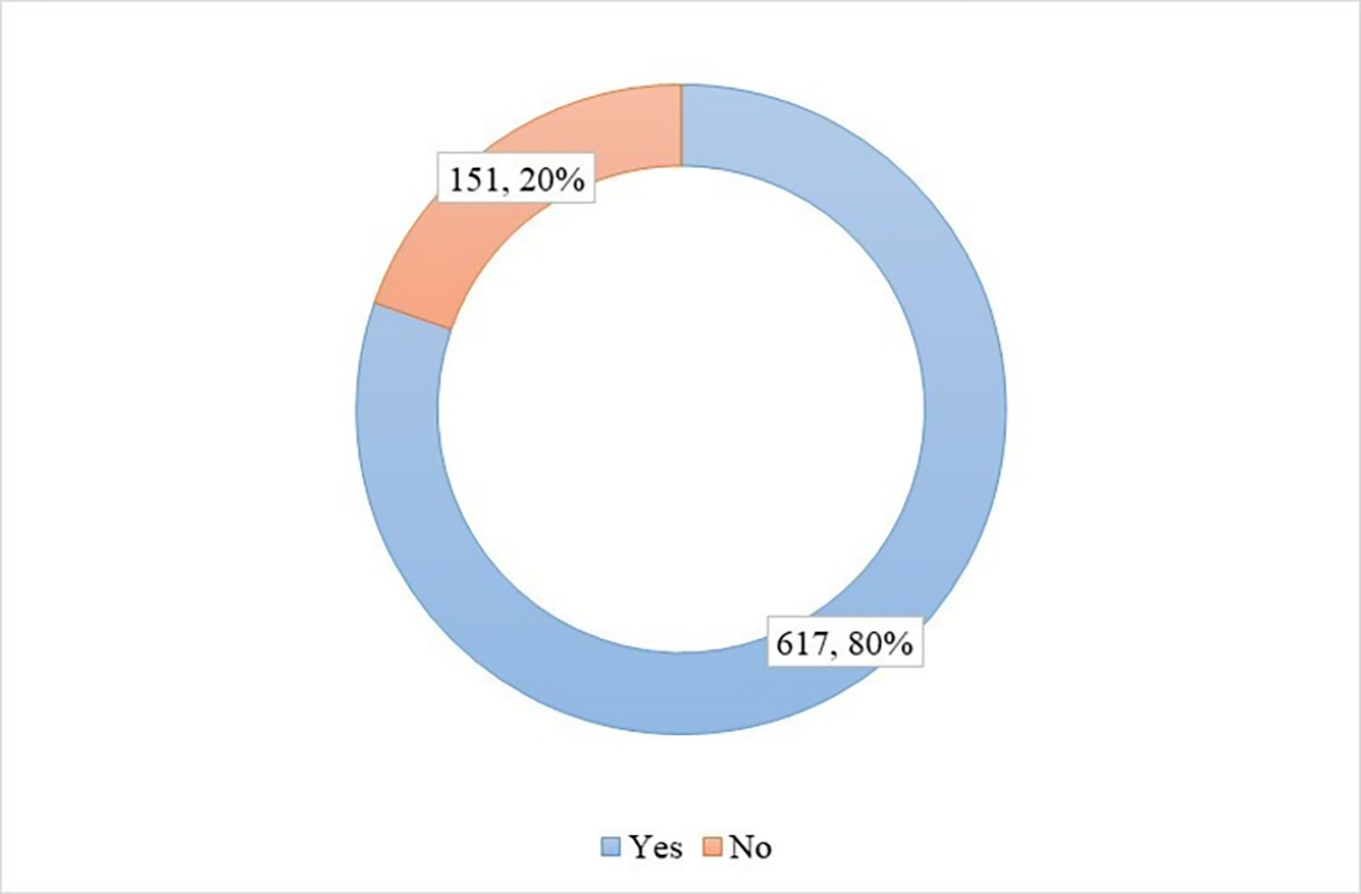
The prevalence of facemask usage among the respondents.

Out of 768 participants, 80% of the participants reported that they wore face masks when they went outside in crowded and public places, while 20% reported that they did not wear face masks (Fig 3).

Among 151 participants with face mask non-adherence, 81 were males and 70 were females. Majority of the participants were of age less than and equal to 30 years (84.1%) and the mean age of the study participants was estimated as 25.05±5.93 years (Range: 18–45 years). Out of 151, 39.7% participants were Urdu ethnicity, 19.9% were Punjabi, 18.5% were Sindhi, 11.3% were Pathan, 2.6% were Baloch, and 7.9% were from other minor ethnicities. About 87.4% of the respondents reported that they lived in urban area. Most of the participants were Muslims (90.1%) and 9.9% were non-Muslims. Out of 151, 48 participants were graduates and 68 were post-graduate. Majority of the participants were unemployed (53%), followed by full-time employed (37.7%), part time employed (5.3%) and housewife (4%) respectively. Of 151 participants, 128 were unmarried, and 23 were married (Table 1).

**Table 1:** Demographic information of study participants with face mask adherence (n = 151).

Variables	n (%)
**Age groups**	
≤30 years	127 (84.1)
>30 years	24 (15.9)
Mean±SD	25.05±5.93
**Gender**	
Male	81 (53.6)
Female	70 (46.4)
**Ethnicity**	
Urdu speaking	60 (39.7)
Sindhi	28 (18.5)
Punjabi	30 (19.9)
Pathan	17 (11.3)
Balochi	4 (2.6)
Others	12 (7.9)
**Locality**	
Urban	132 (87.4)
Rural	19 (12.6)
**Religion**	
Muslim	136 (90.1)
Non-Muslim	15 (9.9)
**Education level**	
Primary	0
Secondary	10 (6.6)
Post-secondary	25 (16.6)
Graduate	48 (31.8)
Post graduate	68 (45)
**Employment status**	
Unemployed	80 (53)
Part time	8 (5.3)
Full time	57 (37.7)
Housewife	6 (4)
**Marital status**	
Single	128 (84.8)
Married	23 (15.2)

Those 20% (n = 151) participants who reported non-compliance with the facemask to prevent COVID-19 spread were then further asked for possible perceived barriers. About 32.4% (SA 13.9%, A 18.5%) of the responders agreed the statement that mask cannot protect them from COVID-19, 13.9% (SA 10.6%; 3.3%) agreed with the statement that they do not need to wear mask because they have already been infected with COVID-19, and almost 35.1% (SA 27.2%; A 7.9%) of the responders agreed that they don’t need to wear mask because they have a strong immune system. About 82.1% (SA 45%; A 37.1%) of the participants agreed that they experience difficulty in breathing when they wear a facemask, 67.6% (SA 40.4%; A 27.2%) of the participants agreed that facemask cause skin problems and almost half of the respondents (SA 27.8%; A 23.2%) agreed that facemask causes stress. Approximately 89.4% (SA 59.6%, A 29.8%) agreed that face mask is too hot in the summer, 27.1% (SA 18.5%; A 8.6%) agreed that face masks make their glasses foggy, 84.1% (SA 60.3%; 23.8%) agreed that wearing a face mask makes them feel uncomfortable. Out of 151 participants, 76.9% agreed (SA 32.5%; A 44.4%) that facemask causes difficulty in eating, drinking and speaking. About 59% of the respondents agreed (SA 41.1%, A 17.9%) that face masks make them look ugly and 43.7% (SA 12.6%; A 31.1%) agreed that face masks hide their smiles. While 30.4% (SA 11.9%; A 18.5%) of the participants agreed that face masks muffle their voices. About 19.2% (SA 11.3%; A 7.9%) of the respondents agreed that people treat them differently if they wear a face mask, whereas 23.6% (SA 13.2%; A 10.6%) agreed that if they wear a face mask then people think they are infected with COVID-19. About 8.3% of the participants agreed (SA 1.3%; A 7.3%) that their family does not support them in wearing a mask and only 4% of the participants agreed (SA 0.7%; A 3.3%) that they do not want to wear a face mask because their friends do not like it. Only 7.3% of the participants agreed that they do not want to wear a mask because it is against their cultural or religious values. Only 9.2% of the participants agreed (SA 7.9%; A 1.3%) that they do not wear face masks because there is no policy regarding it in their locality. Of the respondents, 21.2% (SA 15.2%; A 6%) agreed that it is not necessary to wear face masks because their health consultant does not recommend them (Table 2).

**Table 2:** Descriptive statistics of perceived barriers to wearing face masks.

Perceived barriers	Items	Responses					Mean	St. Dev
		SD	D	N	A	SA		
**Perceived risk**	**It cannot protect me from COVID-19**	13 (8.6%)	63 (41.7%)	26 (17.2%)	28 (18.5%)	21 (13.9%)	2.87	1.22
	**I am already being infected with COVID-19**	35 (23.2%)	70 (46.4%)	25 (16.6%)	5 (3.3%)	16 (10.6%)	2.32	1.18
	**I have strong immune system**	23 (15.2%)	64 (42.4%)	11 (7.3%)	12 (7.9%)	41 (27.2%)	2.89	1.48
**Health concerns**	**It causes me difficulty in breathing**	-	18 (11.9%)	9 (6%)	56 (37.1%)	68 (45%)	4.15	0.98
	**It causes skin problems (i.e. itching, acne and pimples)**	22 (14.6%)	17 (11.3%)	10 (6.6%)	41 (27.2%)	61 (40.4%)	3.68	1.46
	**It causes stress**	33 (21.9%)	30 (19.9%)	11 (7.3%)	35 (23.2%)	42 (27.8%)	3.15	1.55
**Comfort**	**It’s too hot in summer**	-	4 (2.6%)	12 (7.9%)	45 (29.8%)	90 (59.6%)	4.46	0.76
	**It makes my glasses foggy**	71 (47%)	19 (12.6%)	20 (13.2%)	13 (8.6%)	28 (18.5%)	2.39	1.57
	**I feel uncomfortable**	3 (2%)	9 (6%)	12 (7.9%)	36 (23.8%)	91 (60.3%)	4.34	0.99
	**It causes difficulty in eating, drinking and speaking**	2 (1.3%)	11 (7.3%)	22 (14.6%)	67 (44.4%)	49 (32.5%)	3.99	0.94
**Social influences**	**It makes me look ugly**	25 (16.6%)	26 (17.2%)	11 (7.3%)	27 (17.9%)	62 (41.1%)	3.50	1.56
	**It hides my smile**	18 (11.9%)	32 (21.2%)	35 (23.2%)	47 (31.1%)	19 (12.6%)	3.11	1.22
	**It muffles my voice**	40 (26.5%)	45 (29.8%)	20 (13.2%)	28 (18.5%)	18 (11.9%)	2.60	1.37
	**People treat me differently**	44 (29.1%)	61 (40.4%)	17 (11.3%)	12 (7.9%)	17 (11.3%)	2.32	1.28
	**People think I am infected with COVID-19**	54 (35.8%)	51 (33.8%)	10 (6.6%)	16 (10.6%)	20 (13.2%)	2.32	1.40
	**My family does not support it**	94 (62.3%)	30 (19.9%)	14 (9.3%)	11 (7.3%)	2 (1.3%)	1.66	1.01
	**My friends do not like it**	46 (30.5%)	82 (54.3%)	17 (11.3%)	5 (3.3%)	1 (0.7%)	1.89	0.78
**Cultural/religious norms**	**It is against my cultural or religious norms**	122 (80.8%)	10 (6.6%)	8 (5.3%)	11 (7.3%)		1.39	0.89
**Social protocols and health recommendations**	**There is no policy regarding it in my locality**	89 (58.9%)	48 (31.8%)	-	2 (1.3%)	12 (7.9%)	1.68	1.12
	**My health consultant does not recommend me**	81 (53.6%)	24 (15.9%)	14 (9.3%)	9 (6%)	23 (15.2%)	2.13	1.50

Table 3 displays the relationship between potential factors and the perceived barriers to wear facemask The participants of age ≤30 years highly agreed about social protocols and health recommendations as perceived barriers than participants of age >30 years. Hence, statistically significant relationship was found between age and social protocols and health recommendations with p-value = 0.015. Females highly agreed that health concerns (p = 0.031) and social influences (p = 0.001) are the perceived barriers in wearing of facemask as compared to males. With respect to ethnicity, Punjabi and Urdu speaking participants greatly agreed that social influences are the perceived barriers in wearing of facemask. There was statistically significant effect of ethnicity on social influences as perceived barrier with p = 0.001, which indicates at least two means are different. Post hoc comparisons (Table 4) using LSD test indicated that mean social influences score for the Urdu speaking was significantly different than Sindhi (p = 0.013), Punjabi (p = 0.047) and other ethnic groups (p = 0.051). Further, mean social influences score for the Sindhi participants was statistically different than Punjabi (p = 0.001), mean social influences score for the Punjabi participants was statistically different than Pathan (p = 0.002), Balochi (p = 0.022) and other ethnic groups (p = 0.002). Participants who were living in rural areas highly agreed that health concerns and comfort are perceived barriers, whereas participants from urban area greatly agreed that social influences are the perceived barriers to wear facemask. Hence, there was statistically significant association between locality and perceived barriers such as health concerns (p = 0.001), comfort (p = 0.007) and social influences (p = 0.017). Muslim participants highly agreed that health concerns are perceived barriers to wear mask, whereas non-Muslim highly agreed that cultural/religious norms are the perceived barriers to wear facemask. Statistically significant difference was found between religion and perceived barriers such as health concerns (p = 0.03) and cultural religious norms (p = 0.001).

**Table 3:** Stratified analysis of perceived barriers with respect to socio-demographic factors (n = 151).

	Perceived risk		Health concerns		Comfort		Social influences		Cultural/		Social protocols and health recommendations	
									religious norms			
Factors	Mean	SD	Mean	SD	Mean	SD	Mean	SD	Mean	SD	Mean	SD
**Age groups**												
≤30 years	2.68	0.99	3.71	0.88	3.81	0.69	2.48	0.64	1.35	0.83	1.98	0.91
>30 years	2.79	0.92	3.39	0.60	3.71	0.43	2.52	0.57	1.58	1.14	1.50	0.71
p-value (Independent t test)	0.583		0.087		0.464		0.776		0.247		0.015*	
**Gender**	** **	** **	** **	** **	** **	** **	** **	** **	** **	** **	** **	** **
Male	2.60	0.77	3.52	0.86	3.79	0.68	2.33	0.58	1.32	0.70	1.87	0.88
Female	2.80	1.16	3.82	0.81	3.81	0.62	2.67	0.63	1.47	1.06	1.94	0.92
p-value (Independent t test)	0.221		0.031*		0.852		0.001*		0.31		0.622	
**Ethnicity**												
Urdu speaking	2.47	1.02	3.72	0.83	3.64	0.81	2.57	0.57	1.33	0.8	2.04	0.97
Sindhi	3.06	1.16	3.58	0.77	4.00	0.49	2.23	0.69	1.43	0.88	2.04	0.83
Punjabi	2.83	0.84	3.69	0.86	3.76	0.48	2.83	0.64	1.17	0.65	1.70	0.81
Pathan	2.80	0.83	3.69	0.77	3.82	0.52	2.28	0.54	1.53	1.07	1.79	0.90
Balochi	2.58	0.50	4.08	0.50	4.38	0.32	2.11	0.38	1.50	1.00	1.88	0.85
Others	2.50	0.64	3.31	1.21	3.98	0.52	2.20	0.40	1.92	1.38	1.58	0.85
p-value(One-way ANOVA)	0.134		0.606		0.063		0.001*		0.227		0.375	
**Locality**												
Urban	2.72	0.99	3.56	0.81	3.74	0.63	2.53	0.62	1.36	0.90	1.88	0.90
Rural	2.51	0.86	4.35	0.79	4.17	0.67	2.17	0.59	1.58	0.77	2.08	0.85
p-value (Independent t test)	0.374		0.001*		0.007*		0.017*		0.324		0.364	
**Religion**												
Muslim	2.72	1.02	3.71	0.79	3.80	0.66	2.51	0.63	1.15	0.50	1.91	0.90
Non-Muslim	2.47	0.43	3.22	1.17	3.77	0.57	2.28	0.55	3.53	0.74	1.87	0.88
p-value (Independent t test)	0.34		0.03*		0.8		0.18		0.001*		0.87	
**Education**												
Secondary	2.67	0.98	3.87	0.92	3.63	0.66	2.49	0.62	1.5	1.08	1.75	0.72
Post-secondary	2.81	1.07	3.89	0.70	3.89	0.52	2.58	0.56	1.44	1.04	1.82	0.88
Graduate	2.77	0.89	3.61	0.85	3.82	0.69	2.40	0.67	1.33	0.75	2.02	0.90
Post graduate	2.60	1.01	3.58	0.88	3.78	0.67	2.51	0.63	1.40	0.90	1.88	0.93
p-value(One-way ANOVA)	0.74		0.35		0.73		0.68		0.94		0.71	
**Employment status**												
Unemployed	2.75	0.86	3.69	0.86	3.81	0.64	2.45	0.65	1.51	1.04	1.84	0.87
Employed	2.63	1.09	3.63	0.84	3.79	0.66	2.52	0.60	1.25	0.65	1.98	0.92
p-value (Independent t test)	0.43		0.67		0.82		0.473		0.07		0.34	
**Marital status**												
Unmarried	2.75	0.98	3.66	0.87	3.8	0.67	2.47	0.64	1.41	0.92	1.92	0.85
Married	2.39	0.91	3.64	0.72	3.78	0.55	2.58	0.54	1.30	0.70	1.83	1.12
p-value (Independent t test)	0.11		0.89		0.89		0.44		0.61		0.65	

*significant at p≤0.05.

**Table 4:** Post hoc comparisons using LSD test for mean social influences score and ethnicity.

(I) Ethnicity		Mean Difference (I-J)	Std. Error	p-value	95% Confidence Interval	
					Lower Bound	Upper Bound
**Urdu speaking**	**Sindhi**	0.33*	0.14	0.01	0.07	0.61
	**Punjabi**	-0.26*	0.13	0.05	-0.53	0.00
	**Pathan**	0.29	0.16	0.07	-0.03	0.61
	**Balochi**	0.46	0.30	0.13	-0.14	1.06
	**Others**	0.37	0.19	0.05	0.00	0.74
**Sindhi**	**Urdu speaking**	-0.33*	0.14	0.01	-0.61	-0.07
	**Punjabi**	-0.60*	0.16	0.00	-0.91	-0.30
	**Pathan**	-0.05	0.18	0.79	-0.41	0.31
	**Balochi**	0.12	0.32	0.70	-0.50	0.75
	**Others**	0.03	0.20	0.89	-0.38	0.43
**Punjabi**	**Urdu speaking**	0.26*	0.13	0.05	0.00	0.53
	**Sindhi**	0.60*	0.16	0.00	0.30	0.91
	**Pathan**	0.55*	0.18	0.00	0.20	0.91
	**Balochi**	0.72*	0.31	0.02	0.11	1.35
	**Others**	0.63*	0.20	0.00	0.23	1.03
**Pathan**	**Urdu speaking**	-0.29	0.16	0.07	-0.61	0.03
	**Sindhi**	0.05	0.18	0.79	-0.31	0.41
	**Punjabi**	-0.55*	0.18	0.00	-0.91	-0.20
	**Balochi**	0.17	0.33	0.60	-0.48	0.82
	**Others**	0.07	0.22	0.74	-0.36	0.51
**Balochi**	**Urdu speaking**	-0.46	0.30	0.13	-1.06	0.14
	**Sindhi**	-0.12	0.32	0.70	-0.75	0.50
	**Punjabi**	-0.72*	0.31	0.02	-1.35	-0.11
	**Pathan**	-0.17	0.33	0.60	-0.82	0.48
	**Others**	-0.10	0.34	0.78	-0.77	0.58
**Others**	**Urdu speaking**	-0.37	0.19	0.05	-0.74	0.00
	**Sindhi**	-0.03	0.20	0.89	-0.43	0.38
	**Punjabi**	-0.63*	0.20	0.00	-1.03	-0.23
	**Pathan**	-0.07	0.22	0.74	-0.51	0.36
	**Balochi**	0.10	0.34	0.78	-0.58	0.77

*. The mean difference is significant at the 0.05 level.

**Fig 4:**
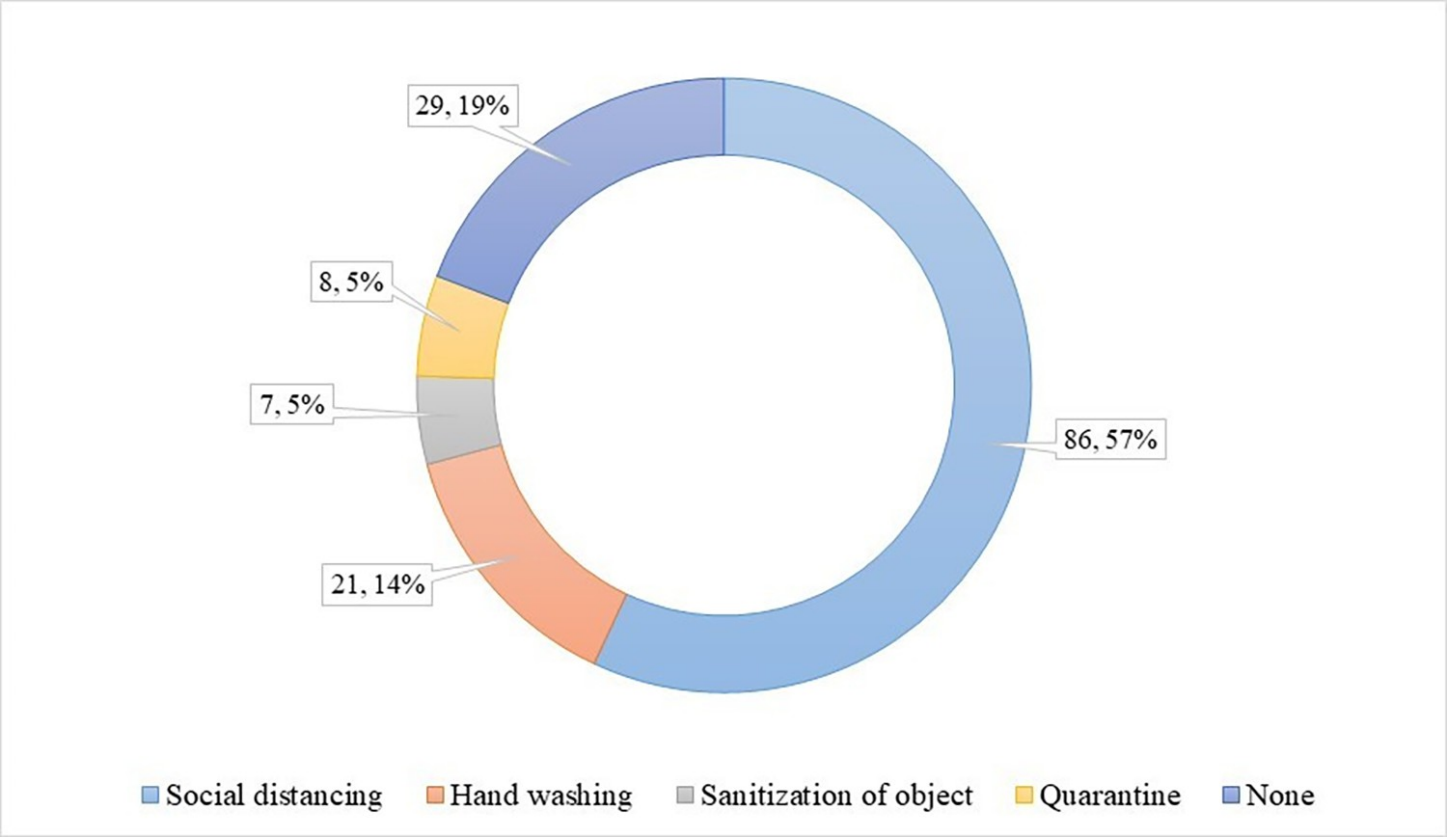
Best alternative to facemask.

151 participants, who reported non-compliance with face masks to prevent COVID-19 spread, were then asked about their opinions regarding the best alternative to face masks. Most of the participants responded that social distancing (57%) is the best alternative to face mask, followed by hand washing (14%) (Fig 4).

## Discussion

In the present study, more than 30% of the participants agreed that masks cannot protect them from COVID-19 and almost 35.1% agreed that they do not need to wear masks because they have a strong immune system. This result can be attributed to the fact that the majority of the respondents (79.3%) were young adults (≤30 years old). Individuals of this age bracket are generally stronger and healthier and may neglect the possibility of getting infected by COVID-19 and believe that precautions should only be taken by vulnerable groups, or outside of their vicinity [21]. A multi-country research also showed that younger population consider themselves at lower perceived risk of contracting COVID-19 than older individuals and therefore low adherence to facemask wearing was observed among these people [22].

No doubt, a face mask is not the most convenient or comfortable protective gear to wear. In excruciatingly hot weather, it can be exhausting as it can cause immense perspiration and the consequent discomfort. It also fogs up the spectacles and therefore causes vision problems and discomfort for those wearing it. Face masks can be uncomfortable if not worn properly and can get in the way of drinking, eating, and speaking (especially on the cellphone). The present study also validates these concerns because it was found that participants highly agreed that feelings of discomfort and health concerns are the most frequent perceived barriers towards wearing face masks. In a previous research, it had been observed that 86% of the respondents agreed that wearing a face mask is uncomfortable [21]. Hence, bad experiences while wearing a mask such as poor ventilation, moisture, thermal issues, and buildup of dirt served as significant barriers in the mask usage [23]. It was also found that mean score for health concerns was significantly higher among females than males (p<0.05). Females tend to be more conscious regarding their skin issues such as acne and rashes and therefore there is a higher health concern found in females than males towards wearing a mask. Furthermore, females also like to be more aware of their overall looks as this serves as a source of self-confidence and therefore, wearing a mask might be effecting their confidence level. There is also a possibility of lack of awareness in people living in rural areas in terms of not having enough knowledge regarding the pandemic and its preventive measures. Another possible reason could be the availability and affordability of face masks. Due to the high demand and hike in price of this protective gear, many people were deprived of its supply and thus people in the rural areas could also have had limited access to face masks, so therefore these people subsided to wearing it less frequently or not wearing it at all.

In the present study, 59% of the participants agreed that social influences such as wearing a face mask makes them look ugly and 43.7% agreed that a face mask hides their smile. While a small proportion (19.2%) of participants agreed that people treat them differently and think that they are infected with COVID-19 if they wear a face mask. These findings might be due to the fact that participants believe that wearing a face mask hides their face and makes people misinterpret their feelings [19, 24]. It has also been observed in previous studies that 75% of the participants agreed that face masks might cause people to view them as ill and therefore they might be subject to consequent discrimination when wearing one [25]. This is an interesting point that individuals want to hide their illness by not wearing facemask in order to avoid critics by others, especially by their friends and colleagues. This is an interesting point because it showcases the mentality of people wanting to hide their illness by not wearing face mask in order to avoid judgement by others, especially by their friends and colleagues. In US, it has been observed that people do not want to wear face mask because they are afraid of being criticized and judged for wearing one, whereas in Asian countries, practice of wearing face masks during viral infection is considered as act of kindness and responsibility towards their society [26]. The results of the present study and other stated experiments suggest that people are more conscious towards the judgement of others and tend to prioritize the criticism over their health.

In the present research, the majority of the participants were Muslims (95.6%), whereas only 4.4% were Non-Muslims. The significantly high mean score for cultural/religious barriers was observed among Non-Muslims than Muslims (p<0.05). This might be due to the fact that for Non-Muslim females, covering of face practices are uncommon and it is against their religious and cultural norms [27]. Another possible justification for such findings can be due to the societal perception that covering one’s face is considered as a conservative act and females who do decide to cover their face are commonly perceived as old-fashioned or highly religious. Therefore, it is a possibility that because of such societal pressures, Non-Muslim females do not want to use face mask as this might put them in the “conservative” category by the community.

Furthermore, in the present research, less proportion of participants agreed that external policy is the perceived barrier to face mask usage. In a previous research, it had been observed that 58% of the participants agreed that it is important to wear face masks because their health experts recommended it. Whereas in this research, it was observed that 21.2% of the participants agreed that it is not necessary to wear face mask because their health consultants do not recommend them, while 69.5% disagreed with this statement [19].

To the best of our knowledge, this was the 1^st^ study that has highlighted and thoroughly assessed the potential barriers perceived by Pakistani community for wearing facemasks to prevent COVID-19 spread. Application of the convenience sampling method was one of the drawbacks of the present research, as it is susceptible to selection bias and lack of generalizability. Online surveys also reduced the generalizability of the findings, since the samples were confined to those with internet connectivity, which therefore restricted certain groups of people (such as illiterate people, people from low socio-economic status, people living in rural areas, people without internet connectivity, etc.) to take part in the study. For further studies, random sampling techniques should be applied as well as people without internet access should be included. This would reduce the sampling error and increase the accuracy of results.

## Conclusion

Despite of the satisfactory facemask adherence, still there are perceived barriers to it. In order to increase utilization of face masks among the general population, strict health policies should be implemented and awareness regarding the importance of face masks should be enhanced by educational interventions.

## Supporting information

S1 File (DOCX)

S1 Data (SAV)
