# Supplementary material for: Perceived barriers to facemask adherence in the covid-19 pandemic in Pakistan-A cross-sectional survey
Source: PLoS One. 2022 May 19;17(5):e0267376. doi: 10.1371/journal.pone.0267376 (PMC9119489; doi:10.1371/journal.pone.0267376)
Supplement: S1 File — (DOCX) [file pone.0267376.s001.docx]

**QUESTIONNAIRE**

This survey designed is to assess your views regarding the new-normal COVID-19 public health interventions i.e. wearing a mask and frequent washing of hands. Participation in this survey is totally voluntary, your data will help us in designing better interventions. All your information and data will be kept secure and only be used by the principal investigator for research purposes.

Name (Optional) ________________

Do you want to participate?

- Yes
- No

Baseline information

Age (years) * _________

Gender *

- Male
- Female

In which area your home is located? *

- Urban
- Rural

Religion*

- Muslim
- Non-Muslim

What is your ethnicity? *

- Urdu Speaking
- Pathan
- Punjabi
- Sindhi
- Balochi
- Kashmiri
- Others

Education level *

- Primary
- Secondary
- Post-secondary
- Graduate
- Post graduate

Employment status *

- Full time
- Part time
- Housewife
- Retired
- Unemployed

Marital status*

- Single
- Married
- Divorced/Separated
- Widow

Do you wear a facemask when you go outside or in public/crowd to prevent/limit COVID-19 spread? *

- Yes
- No

What are the perceived barriers for facemask adherence? Please rate your opinions as;

Strongly agree (SA) “5”, agree (A) “4”, neutral (N) “3”, disagree (D) “2” and to strongly disagree (SD) “1” *

| **Sub-scales** | **Items** | | **Responses** | | | | |
| --- | --- | --- | --- | --- | --- | --- | --- |
| Perceived risk | 1 | It cannot protect me from COVID-19 | ⃣ SD | ⃣ D | ⃣ N | ⃣ A | ⃣ SA |
|  | 2 | I am already being infected with COVID-19 | ⃣ SD | ⃣ D | ⃣ N | ⃣ A | ⃣ SA |
|  | 3 | I have strong immune system | ⃣ SD | ⃣ D | ⃣ N | ⃣ A | ⃣ SA |
| Health concerns | 1 | It causes me difficulty in breathing | ⃣ SD | ⃣ D | ⃣ N | ⃣ A | ⃣ SA |
|  | 2 | It causes skin problems (i.e. itching, acne and pimples) | ⃣ SD | ⃣ D | ⃣ N | ⃣ A | ⃣ SA |
|  | 3 | It causes stress | ⃣ SD | ⃣ D | ⃣ N | ⃣ A | ⃣ SA |
| Comfort | 1 | It’s too hot in summer | ⃣ SD | ⃣ D | ⃣ N | ⃣ A | ⃣ SA |
|  | 2 | It makes my glasses foggy | ⃣ SD | ⃣ D | ⃣ N | ⃣ A | ⃣ SA |
|  | 3 | I feel uncomfortable | ⃣ SD | ⃣ D | ⃣ N | ⃣ A | ⃣ SA |
|  | 4 | It causes difficulty in eating, drinking and speaking | ⃣ SD | ⃣ D | ⃣ N | ⃣ A | ⃣ SA |
| Social influences | 1 | It makes me look ugly | ⃣ SD | ⃣ D | ⃣ N | ⃣ A | ⃣ SA |
|  |  |  |  |  |  |  |  |
|  | 2 | It hides my smile | ⃣ SD | ⃣ D | ⃣ N | ⃣ A | ⃣ SA |
|  | 3 | It muffles my voice | ⃣ SD | ⃣ D | ⃣ N | ⃣ A | ⃣ SA |
|  | 4 | People treat me differently | ⃣ SD | ⃣ D | ⃣ N | ⃣ A | ⃣ SA |
|  | 5 | People think I am infected with COVID-19 | ⃣ SD | ⃣ D | ⃣ N | ⃣ A | ⃣ SA |
|  | 6 | My family does not support it | ⃣ SD | ⃣ D | ⃣ N | ⃣ A | ⃣ SA |
|  | 7 | My friends do not like it | ⃣ SD | ⃣ D | ⃣ N | ⃣ A | ⃣ SA |
| Cultural/ religious norms | 1 | It is against my cultural or religious norms | ⃣ SD | ⃣ D | ⃣ N | ⃣ A | ⃣ SA |
| Social protocols and health recommendations | 1 | There is no policy regarding it in my locality | ⃣ SD | ⃣ D | ⃣ N | ⃣ A | ⃣ SA |
|  |  |  |  |  |  |  |  |
|  | 2 | My health consultant does not recommend me | ⃣ SD | ⃣ D | ⃣ N | ⃣ A | ⃣ SA |
